# Blended learning: how can we optimise undergraduate student engagement?

**DOI:** 10.1186/s12909-016-0716-z

**Published:** 2016-08-04

**Authors:** Caroline E. Morton, Sohag N. Saleh, Susan F. Smith, Ashish Hemani, Akram Ameen, Taylor D. Bennie, Maria Toro-Troconis

**Affiliations:** 1 Medical Education Research Unit, Sir Alexander Fleming Building, Faculty of Medicine, Imperial College London, Exhibition Road, London, SW7 2AZ UK; 2 Faculty of Medicine, 3S1c, Commonwealth Building, Hammersmith Hospital Campus, Imperial College London, Du Cane Road, London, W12 0NN UK; 3 Faculty of Medicine, Sir Alexander Fleming Building, Imperial College London, Exhibition Road, London, SW7 2AZ UK

**Keywords:** Blended learning, E-learning, Medical education, Pharmacology

## Abstract

**Background:**

Blended learning is a combination of online and face-to-face learning and is increasingly of interest for use in undergraduate medical education. It has been used to teach clinical post-graduate students pharmacology but needs evaluation for its use in teaching pharmacology to undergraduate medical students, which represent a different group of students with different learning needs.

**Methods:**

An existing BSc-level module on neuropharmacology was redesigned using the Blended Learning Design Tool (BLEnDT), a tool which uses learning domains (psychomotor, cognitive and affective) to classify learning outcomes into those taught best by self-directed learning (online) or by collaborative learning (face-to-face). Two online courses were developed, one on Neurotransmitters and the other on Neurodegenerative Conditions. These were supported with face-to-face tutorials. Undergraduate students’ engagement with blended learning was explored by the means of three focus groups, the data from which were analysed thematically.

**Results:**

Five major themes emerged from the data 1) Purpose and Acceptability 2) Structure, Focus and Consolidation 3) Preparation and workload 4) Engagement with e-learning component 5) Future Medical Education.

**Conclusion:**

Blended learning was acceptable and of interest to undergraduate students learning this subject. They expressed a desire for more blended learning in their courses, but only if it was highly structured, of high quality and supported by tutorials. Students identified that the ‘blend’ was beneficial rather than purely online learning.

## Background

In the last 20 years, medical education has undergone unprecedented change with a new focus on interactive, more student-centred learning [1–3]. This has been driven to a significant extent by technological innovation [4]. A key part of this evolution has been the increasing use of e-learning or online learning [5, 6]. This has become a key component of medical education as students become more computer literate, computers become more readily available and the demand for technology-based learning at a time convenient to the learner increases [1, 7]. In recent years there has also been increasing pressure on staff resources and space as student numbers have been increased, so moving some learning from the classroom into a more self- directed online learning environment has been employed as one way to cope with this issue. From a learners’ cognitive perspective, it is thought that self-directed learning allows individuals to focus effort on useful information that they do not already know and enhance retention of material [8].

Despite the advances in online technologies it is recognised that as medicine is a practice-based discipline, it is not possible or desirable to fully replace traditionally medical education with online learning [1]. As Khogali asks’ how can internet-based learning advance medical education?’ [7]. Increasingly there is discussion about how both traditional and online teaching can be combined for effective (‘blended’) learning [1, 9]. According to Whitelock and Jelfs [10], blended learning is the combination of tools embedded within an e-learning environment or the combination of a number of pedagogic approaches irrespective of technology used. Littlejohn and Pegler [11] introduced a different approach called ‘blended e-learning’ which shifts the emphasis from the combination of online and face-to-face delivery to the design approach as the main focus. The ‘blend’ aims to find the correct balance between face-to-face and online teaching methodologies. This also integrates well with the idea of a ‘flipped classroom’. This is a concept developed in the 1990s by Walvoord and Anderson which takes much of what is learnt in a group learning environment (i.e. a classroom) and puts it into an individual’s learning space [12]. Individuals learn independently and then come together for dynamic interaction in group sessions [13]. A tool based on this philosophy has been developed at Imperial College London called the Blended Learning Design Tool (BLEnDT) [14]. The pedagogic framework which forms the basis of BLEnDT uses the learning domains (psychomotor, cognitive and affective), in order to classify the verb of each learning outcome an online or face-to-face format [15, 16].

There have been generally positive student opinions (both undergraduate and postgraduate) reported in the small number of studies into blended learning in healthcare education environments, but students’ perceptions varied considerably with the nature of online and face-to-face components, subject content and accessibility to computers [17–22]. These studies took a variety of different approaches to the blend as, unfortunately, the term ‘blended learning’ has been applied to a range of teaching tools and methodologies, from the basic recorded lectures on- line to complex interactive e-modules [9]. There have been no studies to evaluate the teaching of pharmacology to undergraduate medical students through blended learning, although there has been some success with blended learning for teaching pharmacology to nursing students and post-graduate dentists [21, 23].

In the current study, we report students’ views of a blended learning package embedded within a module taken as part of an intercalated BSc in Pharmacology. Staff were interested in reducing the number of lectures within this module and spending more in-class time in interactive sessions. The faculty were interested in using blended learning to create this change and there was also a wish to develop reusable learning resources which could be used with future cohorts and potentially in other programmes.

A qualitative study with an additional questionnaire component was carried out to assess students’ perceptions of the blended module and of blended learning in general. The aims of the study were to address the following questions:

1. What factors optimise undergraduate students’ engagement with blended learning?
2. How do students perceive blended learning?

## Methods

### Design of module

In Imperial College London, all medical students undertake an intercalated BSc year in their 4th year of study during which they specialise in a subject of their choice. Pharmacology is one of the options available and every year about 20 medical students and 5 final year biomedical science students complete this programme. The course is modular, with 3 × 5-week modules followed by a 10-week project. The blended learning course was embedded in the final taught module which primarily deals with neuropharmacology. Prior to this development, the content in this module was largely delivered by lectures with supporting tutorials. The staff were interested in reducing the number of lectures dedicated to the more basic concepts early on in the module so that more in-class time could be dedicated to understanding more complex principles including lecturers’ own research which adds depth to the module. In order to benefit optimally from this rich and complex material the students must be fully comfortable with core concepts.

The module was redesigned using BLEnDT, a tool developed within the School of Medicine in Imperial College London [14]. Readers wishing to use the tool are invited to contact the e-learning team at Imperial College London (elearnm@imperial.ac.uk). However, there are alternative tools available, for example that devised by Laurillard and colleagues [24].

**Fig. 1:**
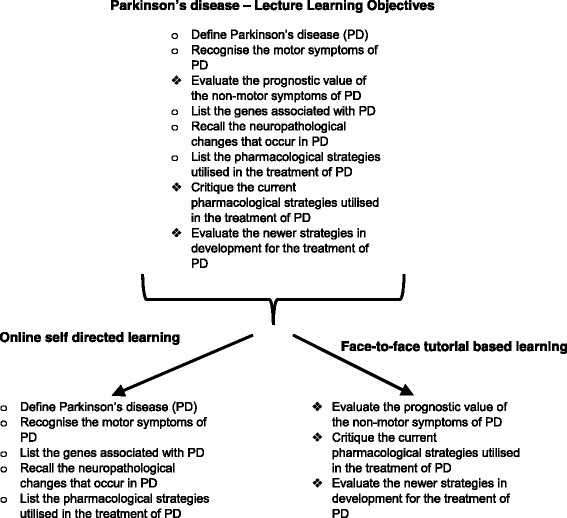
How the learning objectives for Parkinson’s Disease were split into online or tutorial based learning. Core principles were identified as suitable for e-learning, whilst learning objectives requiring the application of principles and the use of higher order thinking skills were covered during the face to face tutorials

**Fig. 2:**
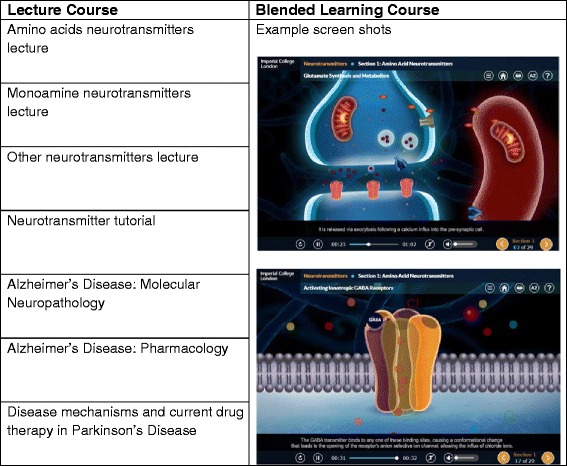
Figure showing how content for lectures was moved into the electronic blended learning course. This figure provides some example screen shots to show core concept lectures were translated into interactive e-learning modules

The learning outcomes of the Neuropharmacology module were analysed using BLEnDT. The split between self-directed (online) and collaborative (face-to-face) learning activities was approximately 40 and 60 % respectively. In order to target the 40 % of the learning outcomes identified as best delivered by self-direction, two online courses were developed: 1) Neurotransmitters 2) Neurodegenerative Conditions. An example of the learning objectives is given in Fig. 1. The online e-learning covered information from 7 × 1 h lectures out of 17 lectures in the module (See Fig. 2). The aim of the blended learning course was not to reduce the number of teaching hours but to reduce the number of hours dedicated to lectures and create time in the programme for discussion of more complex concepts. The total number of hours of teaching did not change. Two blocks of self-directed online learning were developed, each intended to be 2 h in length (however they were made freely available and not time limited) and these were followed by two 90 min tutorials with all 26 students together at the same time.

The courses were developed in HTML5 using graphic reach animations and involved students clicking through a series of screens covering the teaching material which was split into bite-sized “chunks” of knowledge. Animations were used to demonstrate complex sequences of neurotransmitter interactions and interactive quizzes were embedded into the course. The courses were split into small chunks of knowledge following the segmenting principle which suggests the idea of identifying the number of elements or concepts and the interactions between them in order to break complex concepts into small parts and present them sequentially [25]. According to Mayer [26], when a learner receives a continuous presentation combined by several interrelated concepts, the cognitive system becomes overloaded by the demand of too much essential processing. Therefore by using segmenting the learners engage with essential processing without cognitive overload.

The students were timetabled to complete the new online courses on two separate afternoons. Tutorials then followed a week after each course which covered the learning outcomes identified as best delivered in a collaborative way.

### Study design

This study was primarily a qualitative study with a quantitative element to supplement the data collected. It took place at Imperial College London Medical School in Spring 2015. Students completed the self-directed online modules and immediately a 45-minute focus group was carried out with the emphasis on usability of the resource and factors that enhance engagement. These participants (*n* = 14) were convenience sampled from the complete cohort of 26 students (21 medical students who had chosen pharmacology for their BSc option and 5 final year biomedical sciences students). A further two focus groups of 1-hour duration each were carried out after completion of the entire blended module and after students’ end of year exams. All 26 students were invited to participate and 12 took part in these focus groups (*n* = 9, *n* = 3), five of whom had also taken part in the first focus group. Students were provided with lunch but no other incentive was provided to recruit participants into a focus group. Qualitative data was analysed thematically. By the end of the third focus group, saturation of themes was reached. The focus groups were recorded and transcribed by a person (CEM) who had not been involved in the development or teaching of the module or the original design of the research study.

A questionnaire to capture demographics, self-reported computer literacy and students’ satisfaction was distributed for completion online or on paper after the conclusion of the module. It was developed in-house by the research team and incorporated existing questions used in previous questionnaires at Imperial with some additional questions from ‘Usability Evaluation Method for E-Learning courses’ developed by Zaharia and Student Satisfaction Survey form developed by Naaj [18, 27]. The 12 students who took part in the last 2 focus groups completed the questionnaire on paper, and an additional seven students who were unable to attend a focus group completed it online. Questionnaires were analysed with the aid of Stata v14 (StataCorp, Texas). A median score on a 5-point Likert Scale (1 - strongly disagree, 3 - neutral, 5 - strongly agree) was used to demonstrate average students’ scores.

## Results

The questionnaire had an uptake of 73 % (*n* = 19). The mean age of students was 22 years old (SD 1.1) with a roughly even split between the genders (52 % male, *n* = 10). Most of the students were medical students (79 %) with the remainder being final year biomedical science undergraduates. Respondents reported a high level of self-reported computer literacy and a high level of satisfaction with the module (see Table 1).

**Table 1:** Student demographics, computer literacy and satisfaction with the blended learning course

Characteristic	Students (*n* = 19)
Mean age in years (SD)	22 (1.08)
Numbers of males/females	10 males, 9 females
Computer Literacy	Median score on 5-point Likert Scale (Inter Quartile Range [IQR])
I feel confidence using a computer to complete basic tasks	5 (IQR 5–5)
On an average week, I use the internet everyday	5 (IQR 5–5)
Satisfaction	Median score on 5-point Likert Scale (IQR)
Overall I was generally satisfied with the blended learning task	4 (IQR 4–5)
Given a choice I would enrol in another blended learning course	4 (IQR 4–5)
I wish there were more blended learning courses available in my subject area	4 (IQR 3–5)
I would recommend this course to my peers	4 (IQR 4–5)

Student ages are presented as means and standard deviations. Other data are presented as medians and interquartile ranges of responses on a 5 point Likert scale where 5 indicates “Strongly agree” and 1 indicates “Strongly disagree”

Five major themes that emerged from the qualitative element to this study:

1. Purpose and Acceptability
2. Structure, Focus and Consolidation
3. Preparation and Workload
4. Engagement with E-learning component
5. Future Medical Education

### Purpose and acceptability

Blended learning was generally regarded as both acceptable and interesting for students but only if it was highly structured (as discussed below) and delivered in the right way (for example, using high quality animations and interactive quizzes).

Students felt that the e-learning component should not be a replacement for face- to-face teaching but rather should ensure they were prepared for a topic covered in the tutorial and therefore ready to engage in the face-to-face teaching more efficiently. Tutorials were felt to be the key component because they engaged students and allowed instant clarification of questions. The fact that tutorials can be quickly adapted to students’ learning needs was deemed essential.

All students agreed that e-learning was well placed to provide basic knowledge and tutorials helped to form ideas and engage with more complicated processes that required higher level thinking.

*“E-learning to deliver basic knowledge. Tutorials for questions and developing ideas” Student 1*

*“If you have misunderstood something, it [the tutorial] gets you back on track” Student 2*

*“The most efficient approach is to have the e-learning beforehand and then you have a contingency tutorial to check or to ask any questions or to briefly skim over it” Student 3*

*“so often you turn up to a lecture and they jump in so far beyond your knowledge… And you can’t ask effective questions because you don’t know the fundamentals to start with” Student 4*

### Structure, focus and consolidation

Students identified good structure and clear signposting as key for the blended learning modules to work. They noted that the structure of building upwards from simple to complex ideas was logical and made learning points more connected, making for better consolidation and allowing an overview of the whole topic.

Consistent signposting was identified as essential across both the e-learning and tutorial components with ‘core’ and ‘minor’ topics clearly distinguishable. Students reported that this would help them focus on the key learning points since that they found it difficult to distinguish the key topics in a new subject area.

Consolidation was mentioned many times in the context of tutorials consolidating knowledge acquired from the online learning. However, students also reported that they went back to the e-learning at times to clarify points from the tutorial and consolidate that knowledge in greater detail.

There was a high level of satisfaction amongst the students with the interactive components like animations and quizzes, which they reported increased engagement and improved understanding.

Of note, students requested summary revision notes be available at the time of e-learning to allow annotation, which was something that was not provided to them during this project.

*“It’s better to build upwards rather than fill in the gaps” Student 4*

*“Because we had already been exposed to it [the receptors] before in the e-course, when we went over it again it was much easier to understand” Student 5*

*“[the information in e-learning] is all presented equal” Student 2*

### Preparation and workload

Students highlighted concerns that blending courses would increase workload because the e-learning component has background reading incorporated. They admitted that they would have not normally have completed the pre-reading and that by being incorporated into the e-learning they were being forced to do it. They saw the need to signpost ‘core’ and ‘background or minor’ topics as essential to prevent work overload. Students were concerned that lecturers would be too tempted to put extra interesting information into the e-learning because they could and advised faculty to consider blended modules as a whole to ensure that the total content remained a manageable amount of material.

Despite the reservations about workload, students did say that they felt they got more out of the tutorials because they were more adequately prepared and this allowed more efficient learning and effective questions.

*“you need to be careful about how much you put in there because it’s easy to put too much” Student 5*

*“basically consider the content as blended* i.e. *as one” Student 6*

*“if i knew what was kind of going on before I went to things,*

*I would just learn so much more” Student 4*

### Engagement with E-learning component

Students had concerns about the scalability of blended learning to a wider cohort, highlighting that this particular module worked because it is involved a small number of students with a particular interest in the subject matter. There was considerable debate amongst the students about the best ways to ensure students engaged and used the e-learning resource before the tutorials, with many predicting that their peers would not use it to the same degree and therefore would come the tutorial unprepared. Suggestions to improve engagement included tracking individual use of the e-learning course by student ID number, placing an expiry date on access to it and providing timetabled sessions in which to complete the e-learning elements. Other students did not want any restrictions and felt that they should be encouraged to be self-motivated learners.

A number of students wanted the e-learning to be split into small modules of about 1 h in length (the total time for the each e-learning course was 2 h).

*“You’ve got to be responsible for your own actions” Student 7*

*“The problem is, the old age problem, is making sure people do the e-learning beforehand” Student 8*

*“People do take it [the e-learning] less seriously” Student 2*

### Future medical education

Students identified a number of different areas and topics to which they felt blended learning could add value. These included both pre-clinical and clinical topics, although as 4th years they had only completed 1 clinical year. They noted that it would be particularly useful for getting 1st year students to the same level of knowledge and understanding at the beginning of their programme of study. They also suggested a range of other topics particularly those that were taught in fairly short and self contained modules such as renal and musculoskeletal medicine. The formative quizzes were felt to be particularly helpful and they all agreed that they wanted more ways to check their knowledge.

There was some variation in the perceived best purpose for the blended learning. Some students wanted e-learning courses to be available during the first part of every topic, with follow-up tutorials later on. Others felt that they preferred the e-learning to be for relevant supplementary material.

## Discussion

Students had a positive response to the online component reporting that e-learning was highly appropriate to teach basic knowledge which is then extended in tutorials. Students identified that the fact the blend rather than a purely online learning module was beneficial. This is in line with what educationalists already understand about the impact of blended learning to facilitate the ‘flipped classroom’ model [13]. Students can self-teach basic knowledge but need experts for the higher levels thinking skills such as synthesis or evaluation of knowledge [15] which are better delivered by tutorials. The challenge for medical educators is to ensure engagement with the online self-directed component of the module. This initial learning should allow students to fill in the gaps in their knowledge by focusing them on what they do not know which may enhance retention [8]. There is also evidence that learners are often biased in how they select new information to learn [8] and therefore there is often a need for a prescriptive balance between allowing ‘curious’ learners to fill the gaps in their knowledge and dictating what are the most important points within the learning material. This might be less of a consideration with a small cohort where the student to teacher ratio is favourable as was the case in this study and the teacher can highlight gaps in knowledge more easily through tutorials. However there needs to be careful consideration on what impact using self-directed online learning might have with large numbers of students. Care is needed when introducing blended learning on a large scale if, for example, there were plans to use this for the whole student cohort of 320. This is echoed in other studies which have suggested that blended learning can be introduced on the large scale but requires careful planning and organisational change and support [28]. Despite this, students could see the uses of introducing blended learning for some subjects for the entire cohort. BLEnDT provided a structured way to develop the module by analysing clearly defined learning objectives and provided a quantitative split between objectives best taught by self-directed learning and by collaborative learning. This overcame the potential problem of how to structure a ‘blend’.

Educators must be mindful about the amount of content they put into the online component of the blended course. As this project demonstrates and the students made clear, the whole module needs to be considered as one continuous learning experience with an amount of content appropriate to that. Students spent a range of time completing the self-directed work sessions which were designed to be approximately 2-hours in length. We found that students could not differentiate between core topics and background information. This is in line with previous work on this subject [29] and emphasises the need for consistent signposting and structure.

Students requested that detailed notes be made available from the online learning courses as well as the tutorials. This raises an interesting question of how much responsibility students should take for their learning experience. This may have important implications in further online or blended courses of this nature. There is some evidence that re-reading or highlighting pre-fabricated notes is not an effective method of learning [30] and therefore providing detailed notes may not be entirely beneficial. Students might potentially engage more with high level thinking (such as synthesis) when they make their own revision notes [15].

Medical students identified several topics which they felt could be delivered in a blended format across pre-clinical and clinical medicine and suggested that it could be applied to most areas of their medical studies. As discussed before, scaling up for use with large groups of students may present a potential problem. Other studies have suggested that blended learning is ideally place to teach subjects that traditionally have received only a small amount of time within the curriculum, such as dermatology, radiology and ear, nose and throat surgery [31–33]. These topics are also quite visual in nature and may lend themselves to blended learning where interactive images can be incorporated into the online learning component. Students within our study reported an increased engagement and understanding with visual animations on complex pharmacology concepts. This suggests that topics that require students to visualise or recognise 2 or 3 dimensional images or processes could work well with blended learning.

One of the advantages of using a blended course instead of purely online learning is that resources can be updated more easily since tutors can highlight how scientific knowledge has advanced in the tutorials. This is especially true when the online part of the module provides more basic science which is least likely to change, whereas high-level cutting edge ideas can be explored in the tutorial.

There is enormous potential in medical education for more ‘reusable learning re- sources’ to be developed and shared [1]. The new online courses created in this study could be made available for use in other parts of the medical course as well as biomedical science programme, which might reduce the duplication of learning resources. There is also the potential for the sharing of resources between institutions.

A limitation of this study is that this project was relatively small and therefore provides no information about the scalability of blended learning. This will need to be explored in further studies. In addition, this study was performed within an intercalated BSc year with students specialising in pharmacology, who were likely to have a greater than average interest in the subject. This might increase their likelihood of engagement with the blended learning course.

## Conclusion

This paper explores students’ perceptions of blended learning, within one advanced pharmacology module and also within the wider medical school curriculum. It has demonstrated that high quality blended learning is welcomed by undergraduate students in preference to either face-to-face or online alone. The factors that optimise students’ engagement with blended learning are that the e-learning component is highly structured and of high quality and that the blend includes the face-to-face component. They do not want to see complete replacement of didactic teaching with online learning. Careful consideration should be given to the learning design process of blended learning in order to ensure an effective ‘blend’ so design tools such as BLEnDT may help. Careful attention needs to be paid to the structure of the blended learning experience particularly around sign-posting and logical sequence of learning from the basic to the complex. Students were keen to see more blended learning and they perceived blended learning to be acceptable and of interest to undergraduate students.

## Abbreviations

BLEnDT, blended learning design tool; BSc, bachelor of science undergraduate degree; HTML5, hypertext markup language version 5
